# Medical Opinion and Movement

**Published:** 1910-01-08

**Authors:** 


### Medical Opinion and Movement.

FURUNCLES of the External Auditory Meatus, though a simple complaint, are extremely painful and often very obstinate to treatment. Dr. F. Bruch, of Sechenheim, recommends applications of ichthyol. A small pledget of cotton-wool is impregnated with equal quantities of ichthyol and glycerine and introduced into the meatus, followed by a second pledget of dry cotton-wool to prevent any of the solution running out. Care must be taken to prevent the pledgets from exercising any pressure, otherwise the pains would be increased. The application may be renewed once a day, or in very painful cases night and morning, and the treatment should be continued until the desiccation of the furuncle is complete. If the furuncle breaks, the cotton-wool impregnated with ichthyol prevents further infection of the meatus inwards, and there is no fear of setting up eczema of the passage, such as not infrequently occurs with other moist applications or instillations of glycerine and carbolic. In cases in which it may be necessary to incise the furuncle the ichthyol application forms a suitable subsequent dressing.

SOME interesting observations have been carried out on the effect of Alcohol in large and small doses in conditions of Fever by Drs. Dennig and Grünbaum, and an account of them appears in the *Deutsche Archiven für klinische Medizin.* The patients chosen for observation were suffering from pneumonia, typhoid, scarlatina, and other infectious fevers. Blood pressure observations were taken before and at varying intervals after the administration of alcohol in doses of 6 to 35 c.c. of alcohol. Altogether 956 estimations were made on 62 patients. Further observations were also made on animals in a state of fever. As a result these authors find that alcohol in morbid conditions, especially in febrile conditions, has usually a depressing influence upon the blood pressure; very rarely it increased the pressure. Large doses of alcohol have a more marked depressing effect than small, and the blood pressure returns to the original level more rapidly after small doses than after larger doses. Dilatation of the blood vessels plays an important rôle, the fall of blood pressure being due at least in part to the dilatation of the peripheral arteries. From these observations the authors draw the practical conclusion that alcohol should be administered more sparingly in febrile conditions than has hitherto been the custom.

DR. M. F. HESSE draws attention in the *Deutsche Medizinische Wochenschrift* to the importance of examining the fundus of the eye for the differential diagnosis of pernicious anæmia from other forms of severe anæmia—chlorosis, cancerous cachexia, and anæmia secondary to continued hæmorrhage. The author points out that diagnosis of pernicious anæmia is by no means always easy, and the disease is not characterised by a hæmologic formula which is absolutely pathognomonic. More or less severe anæmia caused by repeated hæmorrhage or the presence of a malignant tumour may give rise to blood changes almost or completely identical with those observed in the course of pernicious anæmia. Pernicious anæmia is known to be accompanied by retinal hæmorrhage, but Dr. Hesse draws attention to the fact that these hæmorrhages are almost constant in pernicious anæmia, whereas they are absent in other forms of anæmia, and thus form an important diagnostic sign. Of fifty cases of pernicious anæmia observed in the course of the last twelve years retinal hæmorrhages were present in forty-seven—that is, a proportion of 94 per cent. On the other hand, in fifty-one cases of severe secondary anæmia ophthalmoscopic examination failed to reveal a single retinal hæmorrhage. Similarly, in sixty-four cases of cachectic anæmia due to malignant growths there were no retinal hæmorrhages. Moreover, these retinal hæmorrhages in pernicious anæmia appear to be in some measure indicative of the severity of the disease, and therefore of prognostic value. According to Dr. Hesse, they increase or diminish with aggravation or amelioration of the disease, and their disappearance is frequently one of the first signs of convalescence. In any case, therefore, of suspected pernicious anæmia, examination of the eye should not be neglected. The presence of a retinal hæmorrhage is strongly in favour of this disease, whereas its absence would suggest some malignant tumour, especially cancer of the bowel.

DR. D'HOSTALRICH, at a recent meeting of the Paris Society of Tropical Medicine and Hygiene, called attention to the Lack of Contagiousness of the Leprosy met with in Annam. During the three years during which he has studied the disease in that country, he has not come across a single case from which convincing proof could be drawn of its contagious nature. Moreover, on comparing the smallness of the numbers of lepers met with, as compared with the large number of individuals with whom they live on terms of complete and, to our ideas, incredible promiscuity, it is obvious that the contagium of the disease must be very feeble. The author proposes to devote further time to research on this question. While thinking that contagion is exceptional and difficult, if it exists at all, he is convinced of the importance of heredity as a factor in the dissemination of leprosy.

A CASE in which Tonsillectomy was followed by Basedow's disease has been recently reported by C. J. Koenig to the Société de Laryngologie, d'Otologie et de Rhinologie de Paris. The patient, a young woman of twenty-eight, who suffered from frequent throat-pains, was found to have enlarged tonsils, but no adenoid vegetations. Her general health was good. Tonsillectomy was performed, but four months later the thyroid gland commenced to enlarge, and the patient gradually wasted and lost strength. Later, pains in the stomach, palpitations, tachycardia, and tremor of the hands were noticed. There was, however, no exophthalmos. As the result of rest, massage, and electrical treatment, some degree of improvement ensued, and the gland became reduced in size. The tachycardia persisted, however. Without wishing to attribute the onset of Basedow's disease directly to the tonsillectomy, the author contrasts this case with the numerous examples in the literature of improvement, and even cure, of this disease, which the removal of adenoid vegetations is said to have effected.

An interesting case in which death resulted from Acute Nephritis consequent upon an attempt to procure criminal abortion by means of corrosive sublimate was reported to the Société d'Obstetrique de Paris by Dr. Labourdette. The woman had been given the drug as an intra-uterine injection. Acute nephritis and stomatitis resulted, but there was no suppression of urine, and for a few days before death, no pain. At the autopsy a quantity of pus was found in the peritoneal cavity. In the discussion which followed, Dr. Picqué suggested that the peritonitis had resulted from uterine infection. He had himself seen cases in which healthy tubes allowed pus to escape from the uterus into the peritoneal cavity. Dr. Tissier, on the other hand, maintained that the case was probably one of toxic peritonitis, a condition which, according to medico-legal authorities, is by no means rare.

The dictum of Rosenberger that "Tuberculosis in all its Forms is a Bacteriæmia" has been repudiated by several bacteriologists, though it is supported by Forsyth (*British Medical Journal*, April 24, 1909). One of the most recent researches into the question is that of Dr. Stow, published in the *Medical Record*. He has entered very thoroughly into the matter of technique, and has many criticisms to offer of that adopted by Rosenberger, whose results he believes are due to avoidable contamination. After evolving a routine much more calculated, in his opinion, to eliminate this error, he reports on twenty-eight cases of pulmonary tuberculosis. Of these ten were (clinically) incipient cases, but the sputum of each patient contained acid-fast bacilli. Not one of them showed a single such bacillus in the blood, which was examined with the most minute care. The other eighteen were advanced cases, all with tubercle bacilli demonstrable in the sputum. Of these six showed bacillæmia, twelve did not. Besides these there were twelve autopsy cases, in which blood was removed with the most careful precautions from the heart and from the lungs. Most of them showed tubercle bacilli in the pulmonary blood, but in nine the ventricular blood contained none. In two cases the right ventricle contained bacilli, but not the left; and in the twelfth both ventricles contained them sparsely. Dr. Stow, therefore, declines to agree with Rosenberger's proposition, though he pleads for more work at the problem before it can be considered settled.

The magnificent results which can be obtained by the Applications of Modern Hygiene have seldom been so clearly evidenced as by the story of the recent plague epidemic in San Francisco. In 1907 the disease reappeared in this city, and for some time it made headway in spite of the efforts of sanitary scientists to eradicate it. Curiously, too, it affected the white more than the Oriental population, and the well-to-do quite as much as the poor. A campaign of rat destruction proved ineffective, both against the rats and against the plague: so Blue, the specialist in charge of operations, convened mass meetings of the people in January 1908, at which he explained the measures necessary for combating the epidemic and invited general co-operation. A Citizens' Health Committee was appointed by the Mayor, and hundreds of meetings were held in schools, clubs, factories, and everywhere to get in touch with the people. With characteristic American energy hundreds of thousands of dollars were collected for the work, and 700,000 circulars were distributed. The result has been not only the eradication of plague, but an enormous diminution of all other forms of infective disease. Heart disease now heads the list of fatal diseases in the last annual return. Any more striking proof of the result of a proper observance of the laws of health it would be hard to imagine; would that communities could be induced to carry them out without the stimulus of a serious and deadly epidemic.

Myiasis of the Nasal Cavities is a condition met with but rarely in Britain, but the loathsome nature of the condition and the fact that the patients are usually children render important the knowledge of the best form of treatment. Captain Cameron reports in the *Indian Medical Gazette* his experiments with various parasiticide remedies for the relief of this distressing disease. He finds eucalyptus oil, carbolic acid (one in forty), oil of turpentine, and perchloride of mercury all more or less inefficient, both clinically, and experimentally by immersing living larvæ in them. Chloroform, on the other hand, kills the maggots instantly, and he has adopted in practice a mixture of one part of this substance with two parts of rectified spirit for injection into the nasal cavities by means of a syringe. The remedy causes a painful burning sensation, but it results in a sharp attack of sneezing, with immediate expulsion of quantities of dead larvæ. The cure appears to be permanent and almost instantaneous, and this therapeutic "tip" is one worth bearing in mind.

Some very important researches into the Causation of Beri-beri and its relation to the ingestion of different kinds of rice and other cereals are contributed to the *Archiv. für Schiffs und Tropenhygiene* by Schaumann. The experiments were made upon rabbits, monkeys, cats, dogs, and pigeons, which were fed upon many varying mixtures of rice pap with other foods: the quantities given were always carefully weighed. Many of the diets chosen produced symptoms of paralysis essentially similar to human beri-beri, and nerve degenerations of much the same kind were demonstrated *post-mortem*. Schaumann's conclusions are that beri-beri is probably not due to any infective organism, but that nucleo-proteids are an essential element of animal diet, and that their absence gives rise to degeneration of nerve fibres, polyneuritis, etc. The differences between various forms of rice he attributes mainly to two factors: one, the removal of the endocarp in the process of husking, by which more than half the total nucleo-proteid of the grain is removed; and the other, the action of moulds, which in their avidity for phosphorus split up the nucleo-proteid molecule and destroy it. Old rice is much sought after and esteemed by many of those who live chiefly on this food in the East, and rice is often stored for many years to supply this demand. Such rice is often mould-infected, and outbreaks among lascar crews of sailing ships have been traced also to mouldy rice eaten by the patients.

---

An interesting aspect of Appendicitis as it Complicates the Puerperium was alluded to in this column lately (The Hospital, November 27, 1909, p. 231), in an abstract of a paper by Lapthorn Smith. The view of that author is that many cases of appendicitis during the puerperium are wrongly diagnosed as puerperal sepsis, and that practitioners often suffer undeserved self-reproach through this error. Dr. Palmer Findley, of Nebraska, considers the whole subject of this disease as it affects pregnancy, labour, and the puerperium in a paper based upon seven cases in his personal experience. Only two of these originated in the puerperium, however, and all the patients had had previous attacks. He concludes that pregnancy does not incite a primary attack, though recurrent attacks may be precipitated by pregnancy, labour, or the puerperium. Mild attacks do not alter the course of pregnancy, though severe ones frequently result in the death of the fœtus. A woman of child-bearing age who has had one or more attacks should be operated on, because of the especial liability to recurrence if she become pregnant. Mild attacks during pregnancy may be left until after the puerperium; severe ones should be operated upon at once, and, if gestation is complete or nearly so, it should first be terminated and the appendicectomy then done forthwith. When operating during the course of pregnancy every effort should be made to avoid miscarriage: rest, opiates, and the minimum of manual interference with the uterus contribute to this.

---

The Position of the Apex Beat of the Heart in Children has been very carefully studied by Dr. J. F. H. Sawyer, whose results are recorded in the *British Journal of Children's Diseases.* It has always been recognised by teachers of medicine and in most text-books that the position of the apex beat is distinctly different in the child and the adult, but there is as a rule no very clear indication of the age at which the metamorphosis takes place. To ascertain this examination of 500 children was undertaken in the out-patient departments of the General and Children's Hospitals, Birmingham. The examinations were consecutive, and no effort was made to pick out well-developed children; naturally, therefore, the batch cannot quite be regarded as typically healthy children. No cases of disease of the heart or lungs was included, nor of any other malady which might displace the heart. All examinations were made in the erect posture. For some reason not explained those with apex beats in the fourth left space were excluded; this seems a pity, as this phenomenon is not at all uncommon in children, and it would be of interest to know its exact frequency. The position of the nipple was adopted as the criterion rather than the mid-clavicular line, owing to the difficulty of defining the latter in children. In the first year of life the beat is outside the nipple in two-thirds of the cases, and within it in 3 per cent. only. In the second year the percentage of apex beats outside the nipple reaches 70, dropping to 50 in the third year. After three years of age it is much more exceptional to see this position, and the proportion diminishes steadily from 18 per cent. in the fourth year to 3 per cent. in the eighth year and nothing at all at puberty. From the end of the second year until the end of the tenth the usual situation (80 per cent.) is the nipple line itself, and even at 14 and 15 the proportion is 30 per cent. These results show that the normal adult beat is attained at a distinctly later period of growth than has been hitherto supposed.

---

A very rare and serious Complication of Scarlet Fever is reported in the *Glasgow Medical Journal* by Drs. Griffiths and Riddell, of the Brook Fever Hospital, London. This is rupture of the vessels of the neck into the pharynx, an accident which they have seen twice in the last two years. The first patient was a boy of nine, suffering from a typical but mild attack of scarlet fever. On the twenty-third day the temperature rose to 101.4°, and pain in the right side of the neck was complained of. Next day the temperature was lower, though enlarged glands were noted. Three days later there was much tender œdema of the neck, with swelling of the right tonsil and blood-stained discharge from the nose. Later still a swelling was incised externally, and under slight pressure a cavity was opened which communicated with the jugular vein: hæmorrhage ensued and caused death in about a minute. In the other case it was the internal carotid which gave way. A convalescent child was sitting propped up in bed eating rice pudding when he coughed arterial blood in such quantities as completely to saturate the bed, and died at once. In both these cases the condition of the lymphatic glands suggested that the sequence of events was the liquefaction of one or more of them followed by ulceration into the vessel and subsequently into the nasopharynx and pharynx respectively. In neither case were there any signs which might at the time have suggested the implication of any of the blood-vessels of the neck or the extremely rapid fatal result. It is also to be noted that in the second case an examination of the pharynx was made only an hour before the fatal hæmorrhage, and no signs of ulceration were detected. It is evident that in such cases, which are fortunately rare, no treatment is likely to be of any avail.

